# The expanding application of antisense oligonucleotides to neurodegenerative diseases

**DOI:** 10.1172/JCI186116

**Published:** 2024-10-01

**Authors:** Charlotte J. Sumner, Timothy M. Miller

**Affiliations:** 1 Departments of Neurology, Neuroscience, and Genetic Medicine, Johns Hopkins University School of Medicine, Baltimore Maryland, USA.; 2 Department of Neurology, Washington University School of Medicine, St. Louis, Missouri, USA.

Despite investment of billions of research dollars, neurodegenerative diseases have been largely resistant to therapeutics development because of incomplete understanding of molecular basis of disease, paucity of disease proximal targets, the complexity and relative inaccessibility of the nervous system, and lack of drug technologies that can directly target known genetic causes of disease. In recent decades, the *JCI* has published key preclinical advances highlighting the potential of DNA and RNA targeting therapeutics to be both safely delivered to the CNS and tackle the most proximal causes of neurogenetic disease. Two antisense oligonucleotides (ASOs) are now approved for the treatment of patients with the degenerative motor neuron diseases spinal muscular atrophy (SMA) and familial amyotrophic lateral sclerosis (ALS). Systemically delivered ASOs are also approved for Duchenne muscular dystrophy and familial amyloid neuropathy. Many others are in preclinical and clinical development in hopes that this therapeutic technology can be applied to a multitude of neurological diseases.

## Cerebrospinal fluid–delivered ASOs distribute to the CNS

**Figure 1:**
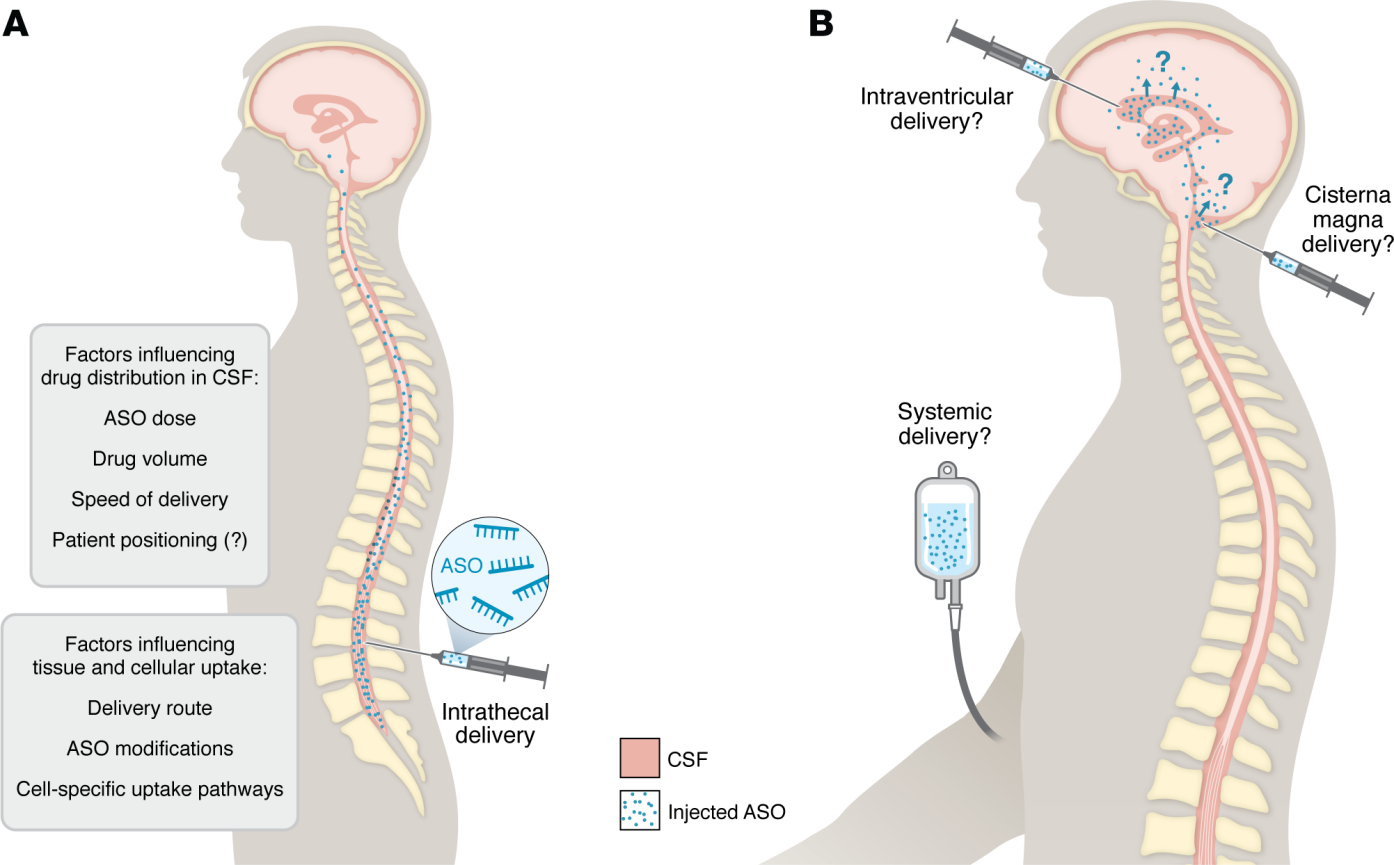
Delivery of ASOs to the CNS for neurodegenerative diseases. (**A**) ASOs are currently administered by lumbar intrathecal injection and diffuse throughout the CSF and into the spinal cord and brain parenchyma. Multiple factors likely influence drug distribution and cellular uptake. Intrathecally-delivered ASOs are currently FDA approved for use in the motor neuron diseases SMA and ALS. (**B**) In the future, with advances in ASO chemistries and delivery technologies, ASOs may be delivered to the lateral ventricle and/or cisterna magna as well as systemically with the capacity to cross the blood-brain barrier.

As synthetic, single-stranded short nucleic acids, ASOs are designed to target a particular pre-mRNA and/or mRNA. After cell surface binding, ASOs are taken up by endocytosis and released into the cytoplasm and nucleus. They bind RNA by Watson-Crick base pairing and can modulate protein production either by degrading their target mRNA or by modulating pre-mRNA splicing or stability. Although first developed in the 1970s, advances in chemical modifications and manufacturing were required to enable the first approved drug for CMV retinitis in 1998. Most ASOs contain a phosphorothioate backbone with one or more 2′ribose sugar or sugar-phosphate modifications, which enhance plasma half-life, resistance to nucleases, target specificity, and tissue uptake. Importantly, as large, negatively charged molecules, ASOs do not cross blood-CNS barriers. A fundamental advance enabling use of ASOs for neurological disease was the demonstration that ASOs can distribute into the spinal cord and brain parenchyma when delivered to the cerebrospinal fluid (CSF) by intracerebroventricular or lumbar intrathecal injection in mice and nonhuman primates (1, 2). Furthermore, the relatively long half-life of ASOs in the CNS enables periodic injections (3). In humans, ASOs are currently delivered by bolus lumbar intrathecal injection, with several loading doses given over the first 2 months followed by dosing every 1–6 months thereafter (Figure 1). Work is ongoing to further understand the pharmacology of ASOs in the CSF (4).

## ASOs are effective in degenerative motor neuron diseases

Developed as part of academic-industry partnerships, nusinersen and tofersen are now in the clinic for patients with SMA and ALS, respectively. The two drugs have distinct molecular actions, illustrating ASO versatility. SMA, historically the leading genetic cause of infant death, is an early-onset disease of α–motor neurons caused by recessive, loss-of-function mutations of the survival motor neuron 1 gene (*SMN1*) (5). Nusinersen targets pre-mRNAs arising from the paralogous *SMN2* gene, which is retained in all patients. Compared with SMN1, the splicing of SMN2 pre-mRNAs is altered by a single nucleotide variant that promotes exclusion of exon 7 and production of truncated, nonfunctional SMN protein. Nusinersen binds the intronic splicing silencer ISS-N1 in SMN2 pre-mRNAs, restoring exon 7 retention and increasing translation of full-length SMN protein. ALS is an adult-onset disease that affects both α– and corticospinal motor neurons, with patients surviving an average of 3–5 years from time of diagnosis (6). Approximately 10% of ALS is due to a known genetic cause, with 10% of this group due to mutations of the superoxide dismutase 1 gene (*SOD1*). Dominant missense mutations of *SOD1* cause a gain-of-function toxic protein that triggers motor neuron degeneration. Tofersen is a “gapmer” designed to bind SOD1 mRNA and recruit RNase-H to degrade the resulting mRNA-ASO duplex, thus lowering SOD1 protein levels (7).

Clinical trials of nusinersen and tofersen were enabled not only by the development of robust, disease-specific motor functional outcome measures, but also by the discovery of biomarkers that assess drug engagement and activity. SOD1 CSF levels allow assessment of target protein knockdown in patients with ALS (8), while serum and CSF neurofilament levels serve as indicators of neurodegeneration (9, 10). When given to symptomatic infants and children with SMA in phase III trials, nusinersen increased motor function and survival and reduced neurofilament levels (9, 11, 12). Even more remarkable benefits were observed when nusinersen was administered to presymptomatic infants with SMA soon after birth (13). Many infants not expected to even gain head control achieved motor milestones similarly to those of normally developing children. Nusinersen was approved by the FDA for all patients with SMA in 2016, and to date, over 14,000 patients have been dosed worldwide. In the 6-month phase III trial of tofersen in symptomatic mutant SOD1 ALS patients, serum neurofilament levels were lowered by 60%, suggesting a substantial slowing of the neurodegenerative disease process. Despite this, tofersen failed to reach significance on the primary outcome of the ALS functional rating scale at 6 months (10). However, during the extension phase after 1 year or more, the clinical benefit became evident, with stabilization in many participants and improvement in strength and breathing parameters in 25%. In a disease with relentless progression, this degree of improvement is striking. Tofersen received accelerated approval by the FDA in April 2023 based on neurofilament lowering likely predicting clinical improvements.

## The challenges facing ASO development for neurodegenerative diseases

Despite unprecedented successes in both SMA and ALS, there have also been noteworthy disappointments. A highly anticipated phase III trial of tominersen, an ASO designed to lower huntingtin mRNA and protein levels in patients with manifest Huntington’s disease, failed to show clinical benefit (14). In another setback for neurodegenerative diseases, two groups tested an ASO that lowers the sense strand of C9orf72 in patients with familial ALS caused by repeat expansion mutations of the C9orf72 gene. Although the ASO showed target engagement with reduction in C9orf72-associated dipeptides, serum neurofilament levels increased consistent with neuronal injury, and there were suggestions of clinical worsening. Finally, lowering of ataxin-2, which has been genetically linked to ALS (intermediate expansions associated with ALS; ref. 15) in a clinical trial of patients with sporadic ALS did not lower neurofilament levels nor improve clinical outcomes.

As with all drug development programs, lack of clinical benefit sparks reinvestigation of the target and patient population as well as drug pharmacokinetics and pharmacodynamics. Regarding the choice of target, there is some concern that lowering of an essential protein may not be tolerated in some cases and thus require allele specific targeting. Biodistribution of ASOs after lumbar intrathecal dosing also remains an ongoing concern, particularly for diseases requiring deep brain tissue penetration. Examination of nusinersen-treated tissues from individuals with SMA at autopsy revealed a caudal to rostral gradient of ASO concentration with the highest concentrations of nusinersen in the lumbar spinal cord but little to no drug in brain stem or brain cortical regions (16). A trial of higher-dose nusinersen in patients with SMA is ongoing (NCT04089566) — the doses of ASOs used in other neurodegenerative disorders are considerably higher, with larger dosing volumes. While higher doses improve brain delivery, they can also heighten risk for drug toxicity, including proinflammatory effects of foreign DNA/RNA recognized by the innate immune system. Although modifications of the ASO backbone and 2′-position and in vitro/in vivo screening are all designed to reduce interactions with the immune system, inflammatory adverse events have been observed in ASO clinical trials, including myelitis and meningitis in some patients with ALS treated with tofersen (10).

A nagging, haunting question in neurodegeneration is whether removing the proximal cause of disease will be enough to reverse or substantially slow disease once it enters a symptomatic phase. In the case of SMA, striking efficacy can be seen when nusinersen is initiated in infants prior to symptom onset, but modest improvements or at best stabilization is observed in older children and adults. Based on these observations, population-wide neonatal genetic screening for SMA was implemented in the United States and increasingly in Europe to enable prompt treatment initiation neonatally. In the case of patients with symptomatic SOD1 ALS, 25% of patients treated with tofersen improve, suggesting that some reversal is possible. An ongoing clinical trial is testing whether more significant improvements can be attained when tofersen is administered presymptomatically (NCT04856982). Whether other neurodegenerative disorders, particularly those with very long prodromal phases, are reversible at a symptomatic stage remains to be determined.

## The future of ASOs for neurological diseases

Advances in drug delivery and chemistry will enable the application of ASO technology more broadly for neurological diseases. Although patients tolerate intermittent intrathecal dosing remarkably well, it is burdensome over a lifetime, particularly for those with structural spine disease, and it can result in postlumbar puncture syndrome. An anchored intrathecal port-catheter device for delivering nusinersen is currently been tested in patients with SMA (NCT05866419). Chemical modifications of ASOs are also improving duration of action and tolerability. For example, a clinical trial is ongoing of a new generation, splice-switching ASO for SMA, which might allow dosing every 9 or 12 months (NCT05575011). Advances in ASO conjugates will also enable improved uptake after systemic dosing to other parts of the nervous system, such a peripheral nerve (17) and skeletal muscle (18) and across the blood brain barrier, enhancing delivery to deep brain structures. Preclinical studies are ongoing in genetic epilepsies (19), spinocerebellar ataxias (20), and prion disease (21). One of the most promising ASOs, MAPT_Rx_, targets the microtubule-associated protein tau (MAPT) mRNA to reduce tau protein, accumulation of which is correlated with Alzheimer’s disease progression. A phase Ib trial in patients with mild Alzheimer’s disease demonstrated a greater than 50% reduction in CSF tau levels (22), and a phase II trial is ongoing (NCT05399888). Another very promising ASO program focuses on reduction of FUS in familial ALS (NCT04768972) (23). In addition to addressing common neurodegenerative disorders, the versatility and ease of manufacturing ASOs could someday enable treatment of individuals with ultrarare neurogenetic disorders that will not attract industry involvement. Academics (24) and a nonprofit foundation (25) are working to determine whether novel, streamlined drug discovery pipelines can be used to safely develop and deliver ASOs to only a single or few individuals. Finally, the future will also undoubtedly bring increasingly complex combinatorial therapies, which merge DNA, RNA and/or protein targeting drugs to achieve increased efficacy.

ASOs are currently transforming the lives of patients with SMA and familial ALS. Despite the ongoing challenges, we are very hopeful that, with continued scientific advances, an increasing number of therapeutic ASOs will stabilize and even reverse disease manifestations in many neurodegenerative diseases.

## Version 1 10/01/2024

Electronic publication
